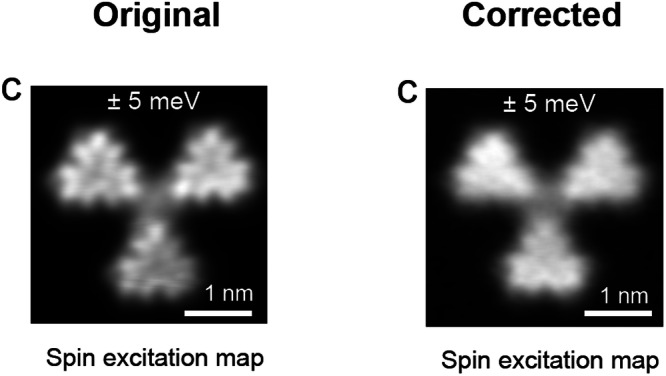# Author Correction: On-surface synthesis of triangulene trimers via dehydration reaction

**DOI:** 10.1038/s41467-025-58331-2

**Published:** 2025-04-01

**Authors:** Suqin Cheng, Zhijie Xue, Can Li, Yufeng Liu, Longjun Xiang, Youqi Ke, Kaking Yan, Shiyong Wang, Ping Yu

**Affiliations:** 1 School of Physical Science and Technology, ShanghaiTech University, 201210 Shanghai, China; 2 Key Laboratory of Artificial Structures and Quantum Control (Ministry of Education), Shenyang National Laboratory for Materials Science, School of Physics and Astronomy, Shanghai Jiao Tong University, 200240 Shanghai, China; 3 Tsung-Dao Lee Institute, Shanghai Jiao Tong University, 200240 Shanghai, China

**Keywords:** Scanning probe microscopy, Synthesis of graphene

Correction to: *Nature Communications* 10.1038/s41467-022-29371-9, published online 31 March 2022

In the version of the article initially published, the image in Fig. 5c was incorrect (the dI/dV mapping image of the Tb trimer was incorrectly used as the dI/dV mapping for the Tt trimer during figure preparation) and has now been corrected in the HTML and PDF versions of the article, as seen in Fig. 1.

Fig. 1 Original and corrected Fig. 5c