# Supplementary material for: On-surface synthesis of triangulene trimers via dehydration reaction
Source: Nat Commun. 2022 Mar 31;13:1705. doi: 10.1038/s41467-022-29371-9 (PMC8971457; doi:10.1038/s41467-022-29371-9)
Supplement: Supplementary file 1 — Supplementary Information [file 41467_2022_29371_MOESM1_ESM.pdf]

Supplementary Information for:
On-Surface Synthesis of Triangulene Trimers via Dehydration Reaction

Suqin Cheng[1,†], Zhijie Xue[1,†], Can Li[2,†], Yufeng Liu[2], Longjun Xiang[1], Youqi Ke[1], Kaking Yan[1], Shiyong Wang[2,3,*] and Ping Yu[1,*]

[1]School of Physical Science and Technology, ShanghaiTech University, 201210 Shanghai, China

[2]Key Laboratory of Artificial Structures and Quantum Control (Ministry of Education), Shenyang National Laboratory for Materials Science, School of Physics and Astronomy, Shanghai Jiao Tong University, Shanghai 200240, China

[3]Tsung-Dao Lee Institute, Shanghai Jiao Tong University, Shanghai, 200240, China

† These authors contributed equally to this work

Contents

1 Synthesis and characterization of precursors 3

1.1 General Methods 3

1.2 Synthetic procedures 3

1.3 NMR and HR-ESI-MS data 6

1.4 X-ray crystallographic data 12

2 Electronic structures of Tb and Tt in the spin states of S = 3 and S = 1 14

2.1 Comparison between MFHM and DFT calculated electronic structures of Tb in spin states of S = 3 and S = 1 14

2.2 Electronic characterization of Tb 15

2.3 DFT calculated electronic structures of Tb and Tt in spin states of S = 3 and S = 1 . 16

2.4 Energy levels comparison between spin states of S = 3 and S = 1 for Tb and Tt . . . 18

2.5 Theorectical simulated LDOS maps of Tb and Tt in the excited spin state of S = 1 . 19

2.6 Constant-height dI/dV maps of Tb and Tt 20

2.7 AFM images of triangulene trimers with additional hydrogen passivation 21

1 Synthesis and characterization of precursors

1.1 General Methods

Most experiments were performed under an atmosphere of dry nitrogen using standard Schlenk techniques. Commercially available reagents were used as received without further purification. 9-(4-bromo-2,6-dimethylphenyl)anthracene was synthesized following literature procedures.[1] The reaction progress was monitored by thin layer chromatography (TLC) which contains silica-coated glass plates and fluorescence marker F254. Crude reaction products were purified by preparative silica gel chromatography (particle size: 45-75 μm, Greagent). NMR spectra were recorded on Bruker AVANCE III HD 500 MHz spectrometer. The NMR measurements were carried out in the liquid-state using deuterated chloroform ($CDCl_3$, 99.9 atom$\%$ D, $\delta_{H-NMR} = 7.26\,ppm$ $\delta_{C-NMR} = 77.3\,ppm$). The peak pattern in ^{1}H-NMR spectra is described by commonly used abbreviations: s = singlet, d = doublet, t = triplet and m = multiplet. ^{1}H and ^{13}C chemical shifts were determined using residual signals of the deuterated solvents or using TMS as the internal standard, and the reported in parts per million (ppm) relative to TMS. High-resolution mass spectrometry (HRMS) was performed on a Q Exactive Focus high resolution instrument (Thermo Fisher, Massachusetts, USA) using electrospray ionization (ESI). The crystal of compound **3** was selected for single-crystal X-ray diffraction. The data was collected on a Bruker APEX-II CCD diffractometer with Mo-Kα radiation (λ = 0.71073). The crystal was kept at 150.0 K during data collection. Using Olex2[2], the structure was solved with the SHELXT[3] structure solution program using Intrinsic Phasing and refined with the SHELXL[4] refinement package using Least Squares minimisation.

1.2 Synthetic procedures

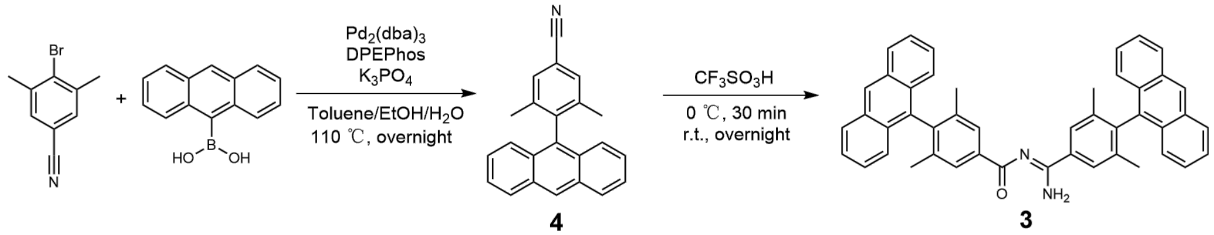

Supplementary Figure 1. The synthetic route of the precursor **3**.

4-(anthracen-9-yl)-3,5-dimethylbenzonitrile (4): A solution of 4-bromo-3,5- dimethylbenzonitrile (1.0 g, 5.0 mmol), anthracene boronic acid (1.3 g, 6.0 mmol), K_3PO_4 (3.2 g, 14.0 mmol), $Pd_2(dba)_3$ (0.4 g, 0.4 mmol) and DPEPhos (0.5 g, 0.9 mmol) was suspended in toluene (40 mL), EtOH (20 mL) and water (10 mL). The suspension was degassed via N_2 sparging for 10 min, then refluxed under N_2 overnight. The reaction mixture was cooled down to room temperature and the solvent was removed via rotary evaporator. The residue was purified by column chromatography (silica gel, heptane/DCM = 5/1) to afford compound **4** as a white solid (0.8 g, 52 $\%$). ^{1}H NMR (500 MHz, $CDCl_3$, 295 K): δ 8.54 (s, 1H), 8.08 (d, *J* = 8.5 Hz, 2H), 7.58 (s, 2H), 7.49 (t, *J* = 7.5 Hz, 2H), 7.39 – 7.36 (m, 2H), 7.30 (d, *J* = 8.5 Hz, 2H), 1.78 (s, 6H); ^{13}C NMR (125 MHz, $CDCl_3$, 295 K): δ 143.30, 139.56, 132.96, 131.51, 131.00, 128.91, 128.86, 127.17, 126.38, 125.44, 124.85, 119.28, 111.56, 19.91; HR-ESI-MS (m/z): $[M]^+$ calcd. for $C_{23}H_{18}N$, 308.1434; found, 308.1419.

(Z)-N-(amino(4-(anthracen-9-yl)-3, 5-dimethylphenyl)methylene)-4-(anthracen-9-yl)-3,5-dimethylbenzamide (3): Trifluoromethanesulfonic acid (1.0 mL, 11.0 mmol) was slowly added to compound **4** (0.4 g, 1.3 mmol) at 0 °C stirred for 30 min. The mixture was further stirred at room temperature overnight. Water (20 mL) was added and then the mixture was washed with MeOH. The residue was purified by column chromatography (silica gel, heptane/DCM = 2/1) to afford compound **3** as a pale yellow solid (25.0 mg, 6 $\%$). ^{1}H NMR (500 MHz, $CDCl_3$, 295 K): δ 8.53 (s, 1H), 8.51 (s, 1H), 8.33 (s, 2H), 8.08 (t, *J* = 8.0 Hz, 4H), 7.99 (s, 2H), 7.50 – 7.42 (m, 8H), 7.38 – 7.34 (m, 4H),1.87 (s, 6H), 1.85 (s, 6H); ^{13}C NMR (125 MHz, $CDCl_3$, 295 K): δ 181.19, 167.36, 142.54, 141.94, 139.01, 138.00, 137.29, 135.13, 134.62, 134.13, 131.57, 129.23, 129.13, 128.81, 128.72, 128.68, 126.78, 126.54, 126.41, 126.07, 125.82, 125.67, 125.36, 125.28, 77.21, 20.30, 20.19; HR-ESI-MS (m/z): $[M]^+$ calcd. for $C_{46}H_{37}N_2O$, 633.2900; found, 633.2905.

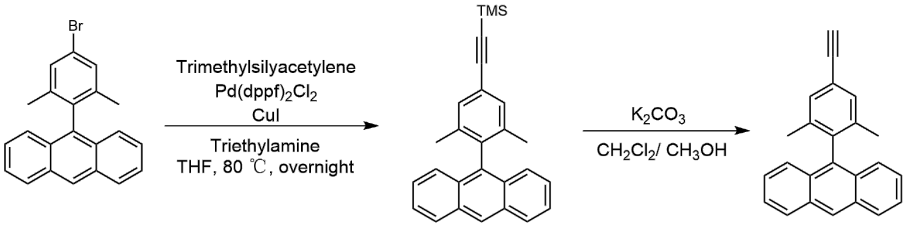

Supplementary Figure 2. The synthetic route of the precursor **6**.

((4-(anthracen-9-yl)-3,5-dimethylphenyl)ethynyl)trimethylsilane (5): 9-(4-bromo-2,6-dimethylphenyl)anthracene (0.2 g, 0.5 mmol), $Pd(dppf)_2Cl_2$ (20.0 mg, 0.02 mmol), Et_3N (15 mL), and CuI (8.0 mg, 0.04 mmol) were dissolved in THF (30 mL). The solution was degassed by passing a stream of nitrogen gas through the solution for 10 min. Trimethylsilylacetylene (0.5 mL, 3.5 mmol) was added and the mixture was stirred at 80 °C overnight. The reaction mixture was cooled to room temperature and the solvent was removed via rotary evaporator. The residue was purified by column chromatography (silica gel, petroleum ether) to afford compound **5** as colourless liquid (0.1 g, 48 %). ^{1}H NMR (500 MHz, $CDCl_3$, 295 K): δ 8.51 (s, 1H), 8.07 (d, *J* = 8.5 Hz, 2H), 7.49 – 7.41 (m, 6H), 7.36 – 7.33 (m, 2H), 1.73 (s, 6H), 0.33 (s, 9H); ^{13}C NMR (125 MHz, $CDCl_3$, 295 K): δ 138.39, 137.98, 134.55, 131.47, 130.91, 129.26, 128.62, 126.41, 125.79, 125.44, 125.19, 122.14, 105.37, 93.76, 19.74; HR-ESI-MS (m/z): $[M]^+$ calcd. for $C_{27}H_{26}Si$, 378.1799; found, 378.1790.

9-(4-ethynyl-2,6-dimethylphenyl)anthracene (6): A solution of compound **5** (70.0 mg, 0.2 mmol) and K_2CO_3 (0.2 g, 1.3 mmol) in DCM (5 mL) and MeOH (5 mL) was vigorously stirred overnight. The solvent was evaporated under vacuum and the residue was purified by column chromatography (silica gel, petroleum ether) to afford compound **6** as a white solid (50.0 mg, 88 %). ^{1}H NMR (500 MHz, $CDCl_3$, 295 K): δ 8.50 (s, 1H), 8.07 (d, *J* = 8.4 Hz, 2H), 7.47 (t, *J* = 6.8 Hz, 2H), 7.43 – 7.41 (m, 4H), 7.38 – 7.31 (m, 2H), 3.13 (s, 1H), 1.73 (s, 6H); ^{13}C NMR (125 MHz, $CDCl_3$, 295 K): δ 138.76, 138.17, 134.50, 131.55, 131.15, 129.31, 128.72, 126.54, 125.91, 125.50, 125.29, 121.23, 83.99, 19.86; HR-ESI-MS (m/z): $[M]^+$ calcd. for $C_{24}H_{18}$, 306.1404; found, 306.1399.

1.3 NMR and HR-ESI-MS data

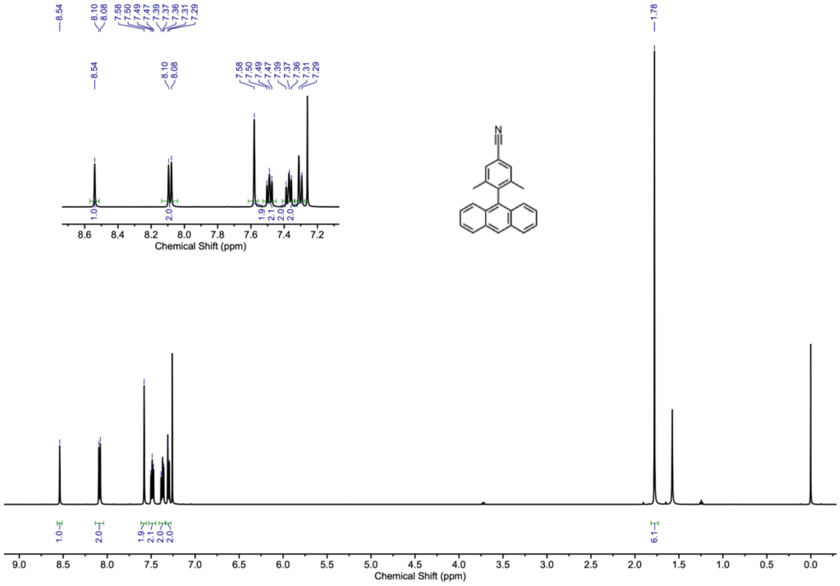

Supplementary Figure 3. ^{1}H NMR spectrum of compound **4** (500 MHz, $CDCl_3$, 295 K).

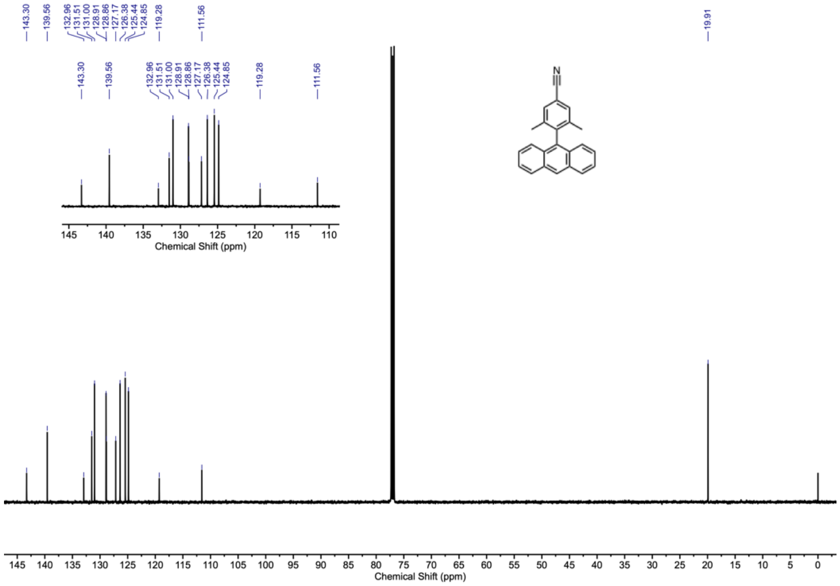

Supplementary Figure 4. ^{13}C NMR spectrum of compound **4** (125 MHz, $CDCl_3$, 295 K).

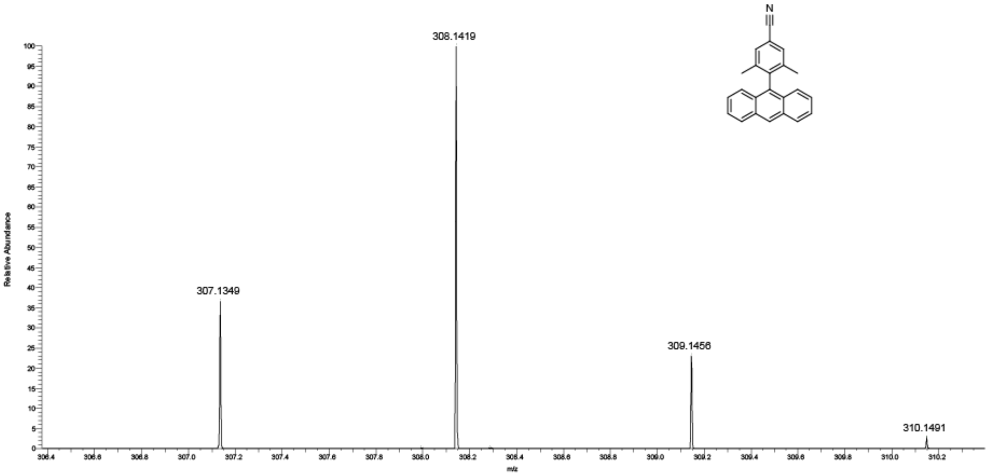

Supplementary Figure 5. ESI-HRMS spectrum of compound **4** (positive mode).

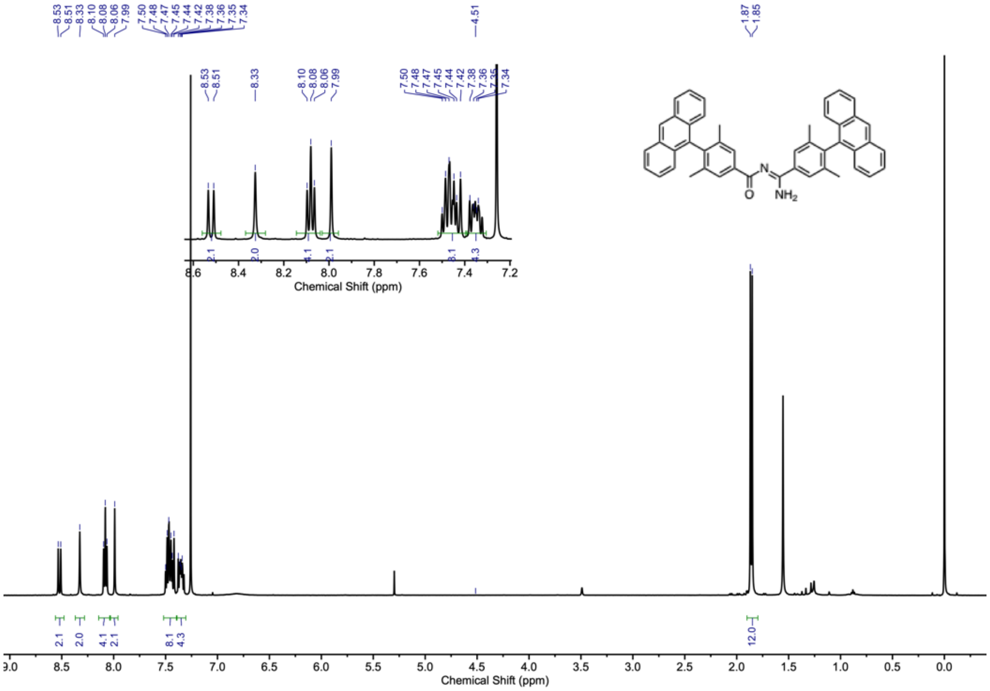

Supplementary Figure 6. ^{1}H NMR spectrum of compound **3** (500 MHz, $CDCl_3$, 295 K).

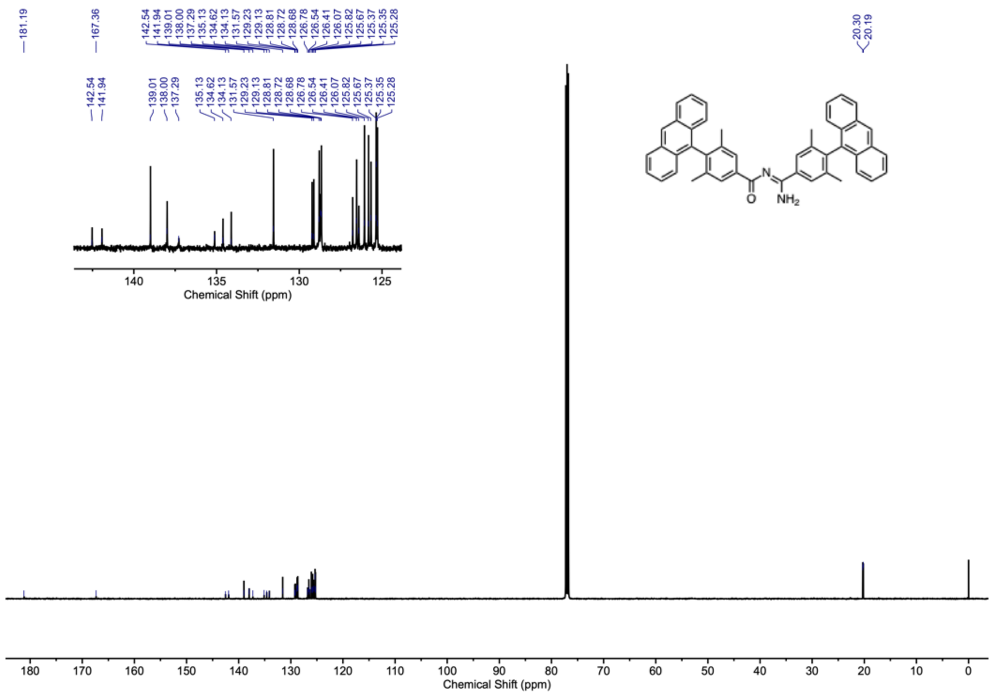

Supplementary Figure 7. ^{13}C NMR spectrum of compound **3** (125 MHz, $CDCl_3$, 295 K).

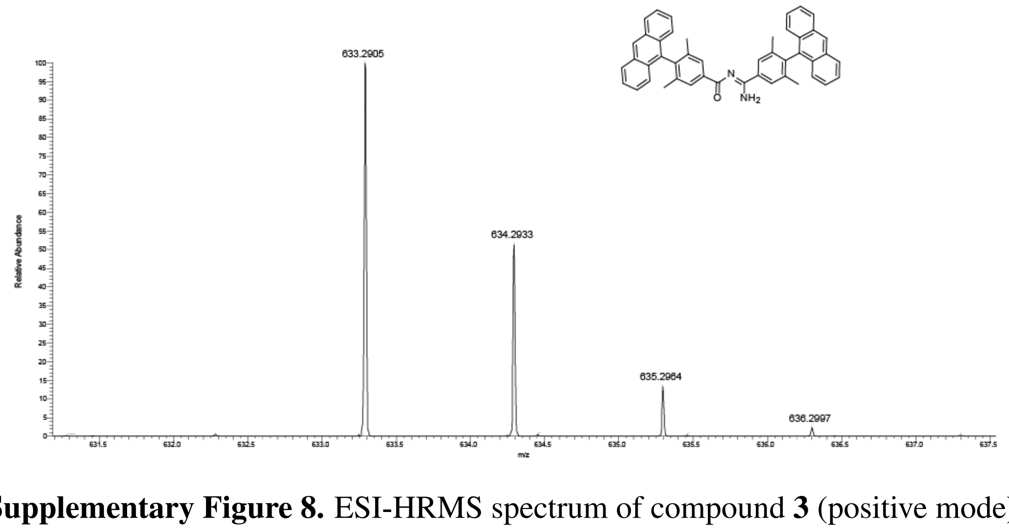

Supplementary Figure 8. ESI-HRMS spectrum of compound **3** (positive mode).

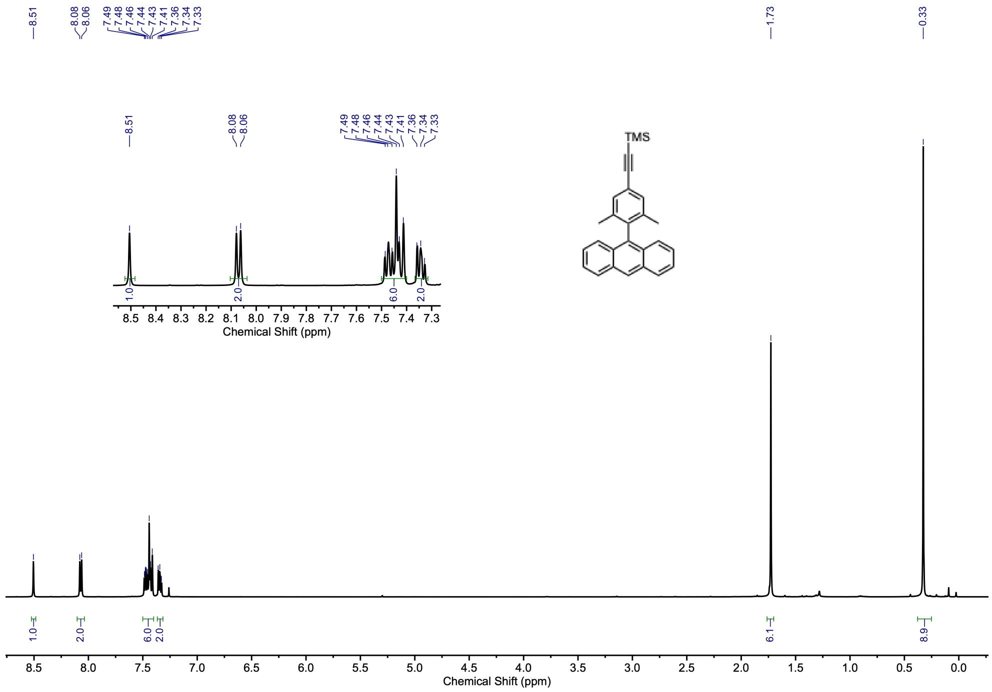

Supplementary Figure 9. ^{1}H NMR spectrum of compound **5** (500 MHz, $CDCl_3$, 295 K).

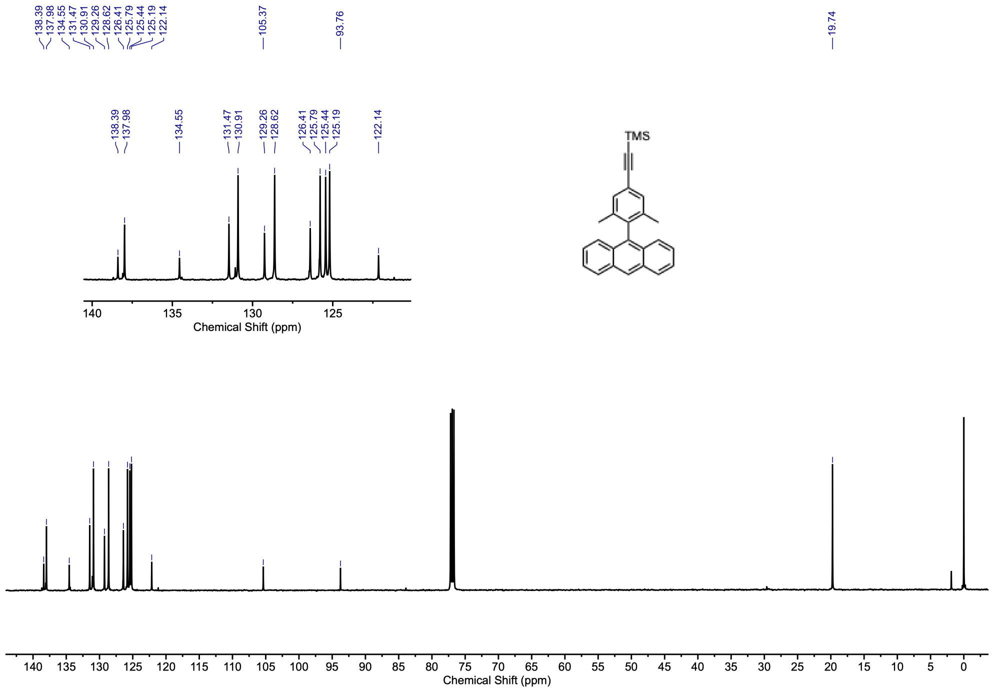

Supplementary Figure 10. ^{13}C NMR spectrum of compound **5** (125 MHz, $CDCl_3$, 295 K).

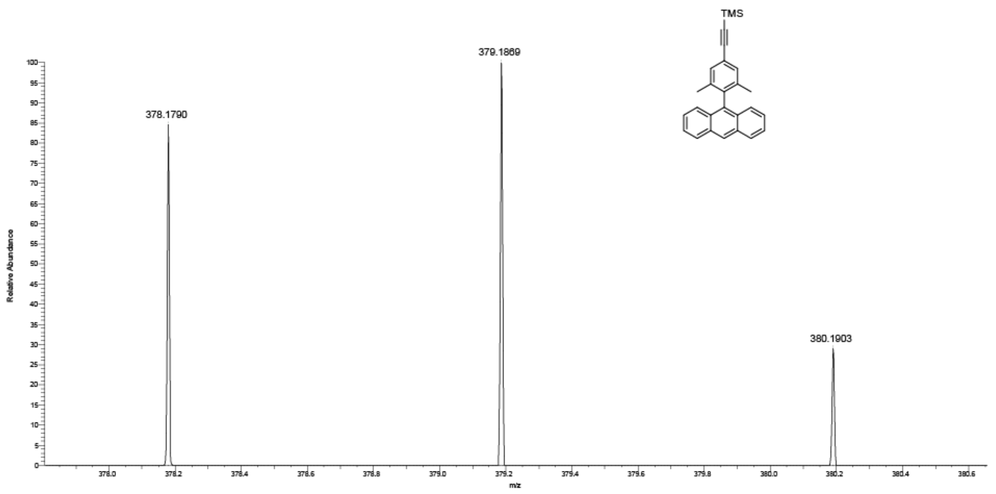

Supplementary Figure 11. ESI-HRMS spectrum of compound **5** (positive mode).

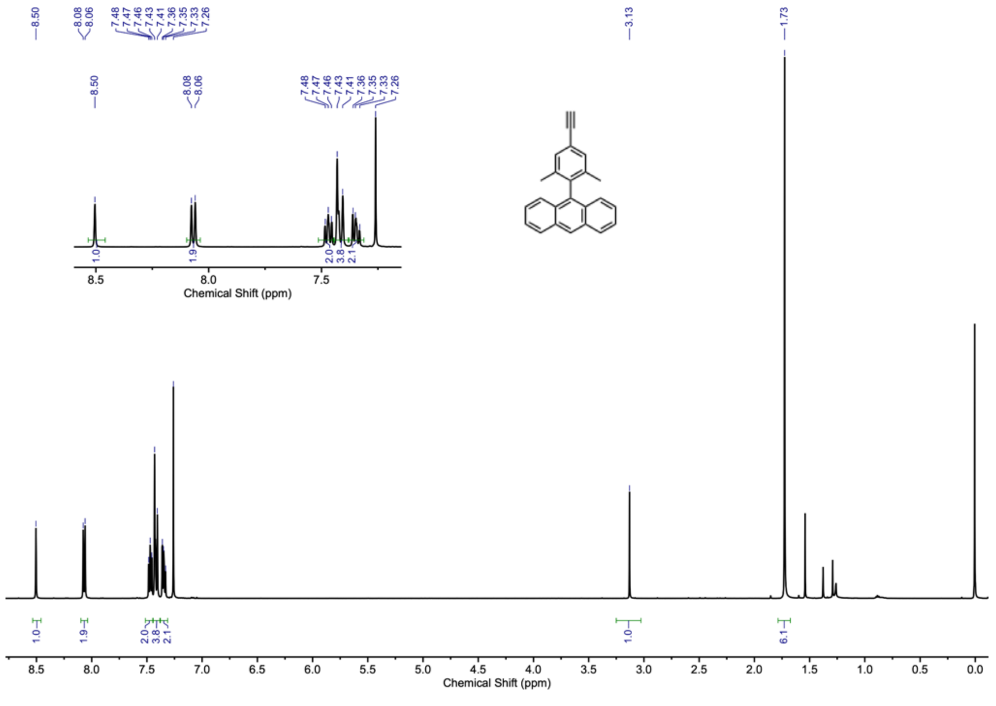

Supplementary Figure 12. ^{1}H NMR spectrum of compound **6** (500 MHz, $CDCl_3$, 295 K).

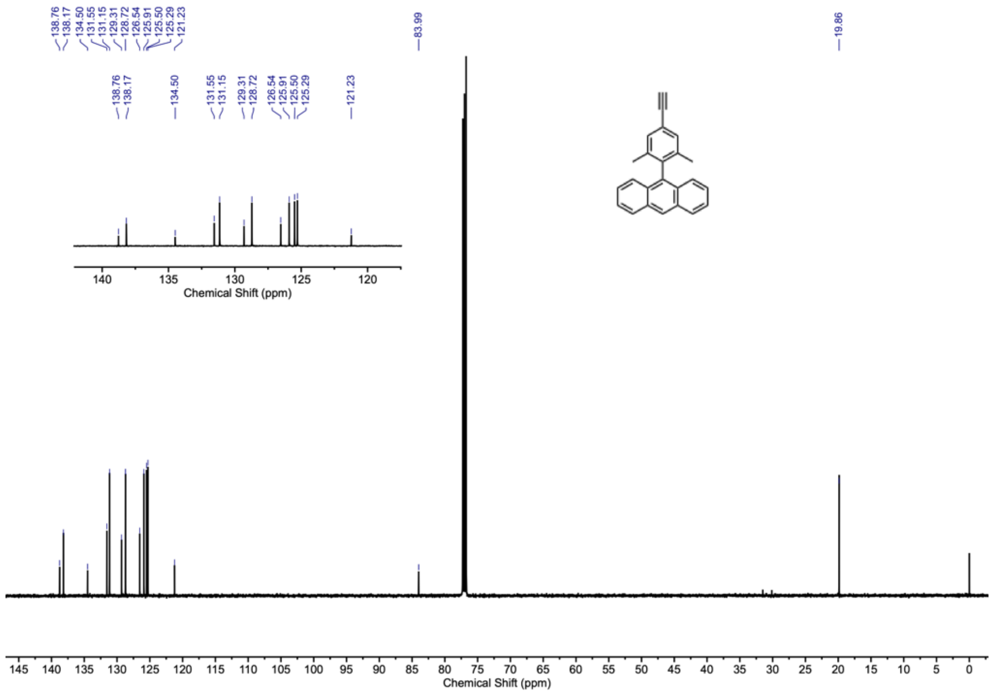

Supplementary Figure 13. ^{13}C NMR spectrum of compound **6** (125 MHz, $CDCl_3$, 295 K).

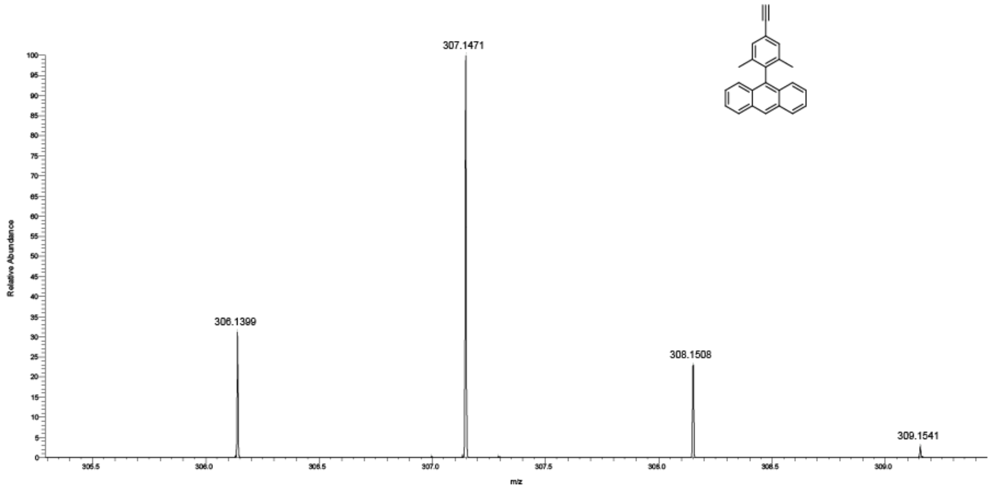

Supplementary Figure 14. ESI-HRMS spectrum of compound **6** (positive mode).

1.4 X-ray crystallographic data

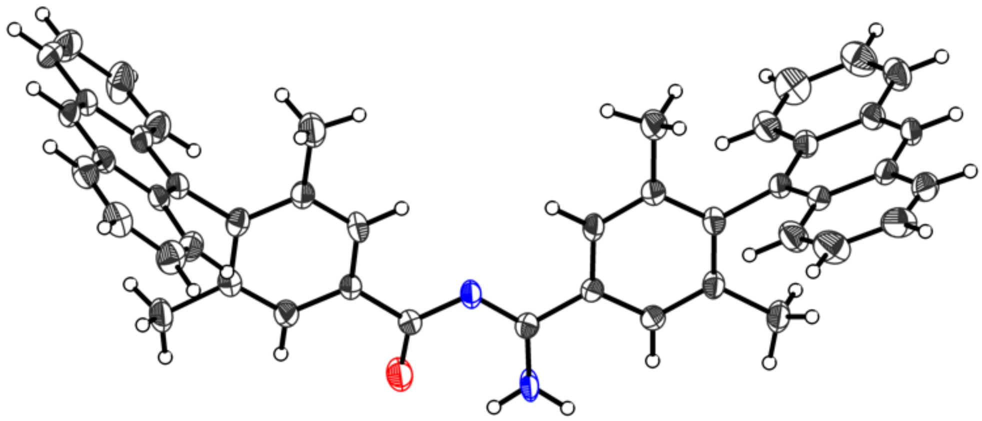

Supplementary Figure 15. X-ray crystallographic structure of compound **3**.

Supplementary Table 1. Crystal data and structure refinement for compound **3**.

Identification code	Compound **3**
Empirical formula	$C_{46}H_{36}N_2O$
Formula weight	632.77
Temperature/K	150.0
Crystal system	triclinic
Space group	P-1
a/Å	11.9407(6)
b/Å	13.3973(8)
c/Å	13.6890(8)
α/°	111.358(3)
β/°	91.793(3)
γ/°	96.495(3)
Volume/Å^3	2020.2(2)
Z	2
$\rho_{calc}\, g/cm^3$	1.040
μ/mm^{-1}	0.062
F(000)	668.0
Crystal size/mm^3	0.15 $\times$ 0.1 $\times$ 0.1
Radiation	MoKα (λ = 0.71073)
2θ range for data collection/°	4.436 to 52.868
Index ranges	-14 $\leq$ h $\leq$ 14, -16 $\leq$ k $\leq$ 16, -17 $\leq$ l $\leq$ 17
Reflections collected	46404
Independent reflections	8255 [R$_{int}$ = 0.1152, R$_{sigma}$ = 0.0731]
Data/restraints/parameters	8255/0/454
Goodness-of-fit on F^2	1.026
Final R indexes [I$\geq$2σ (I)]	R$_1$ = 0.0714, wR$_2$ = 0.1721
Final R indexes [all data]	R$_1$ = 0.1184, wR$_2$ = 0.1935
Largest diff. peak/hole / eÅ^{-3}	0.28/-0.30

2 Electronic structures of Tb and Tt in the spin states of S = 3 and S = 1

2.1 Comparison between MFHM and DFT calculated electronic structures of Tb in spin states of S = 3 and S = 1

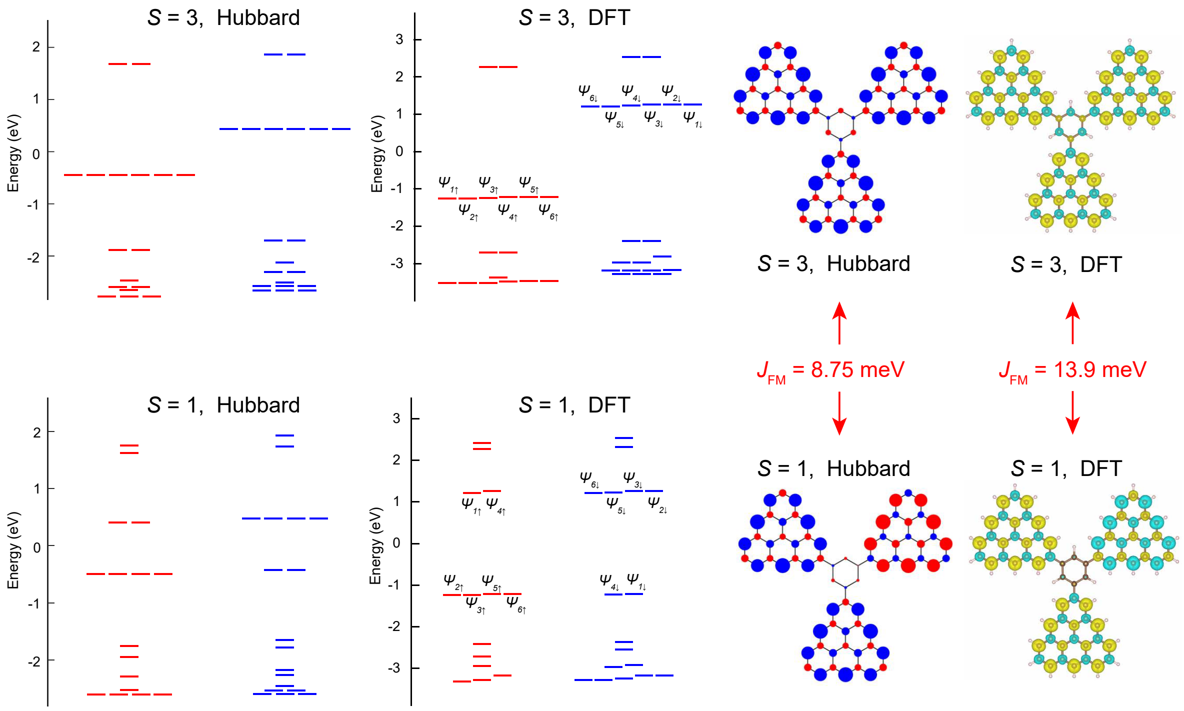

Supplementary Figure 16. MFHM and DFT calculated spin-resolved energy levels of Tb with S = 1 and S = 3. Spin density distributions are shown beside correspondingly. Mean-field Hubbard model and spin-polarized density functional theory calculated spin density distributions: red/green, spin up; blue/yellow, spin down.

2.2 Electronic characterization of Tb

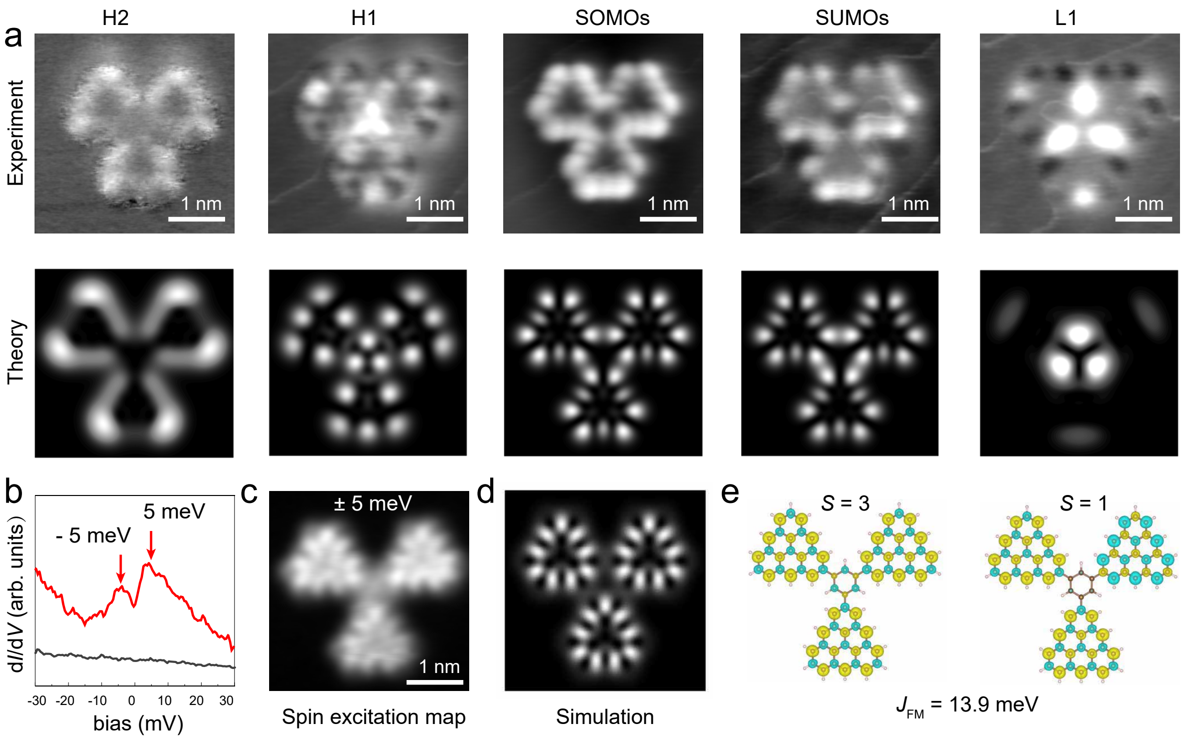

Supplementary Figure 17. Experimental and calculated dI/dV maps of Tb. (a) Constant-current dI/dV maps (top) and theoretical simulated maps (bottom) at energies of -2.1 V (H2), -1.4 V (H1), -0.58 V (SOMOs), 0.93 V (SUMOs) and 1.8 V (L1). (V_{rms} = 25 mV, I = 200 pA). (b) Low-energy dI/dV spectra on Tb. (c,d) Constant-height dI/dV map (c) and theoretical simulated map (d). (e) DFT calculated spin density distribution of Tb in spin state of S = 3 and S = 1. The colors green and yellow represent the spin density distributions. Set point: (b) V = 30 mV, I = 200 pA; (c) V_{rms} = 2 mV.

2.3 DFT calculated electronic structures of Tb and Tt in spin states of *S* = 3 and *S* = 1

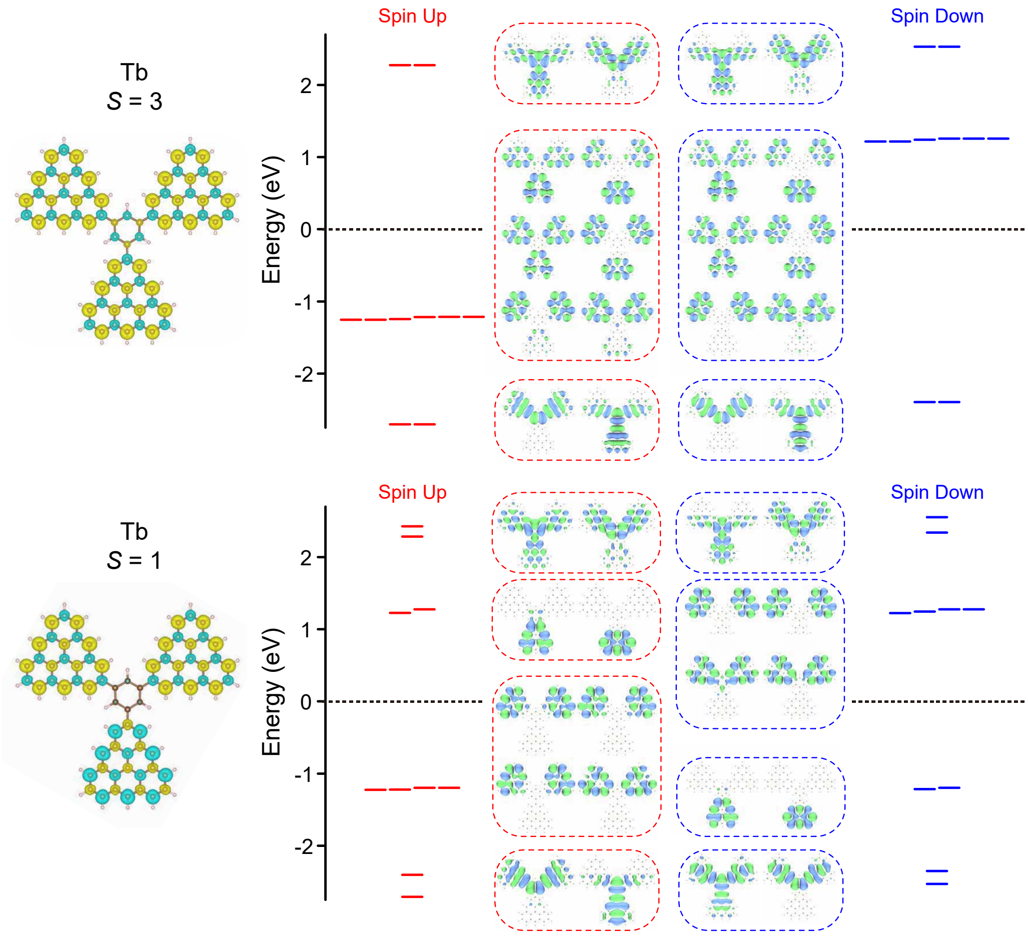

Supplementary Figure 18. DFT calculated spin-resolved energy levels of Tb with *S* = 1 and *S* = 3. The calculated spin density distributions are denoted as color green and yellow for spin up and spin down respectively. DFT calculated wave functions of corresponding molecular orbitals are shown beside. The blue and green isosurface colors indicate opposite phases of the wave function.

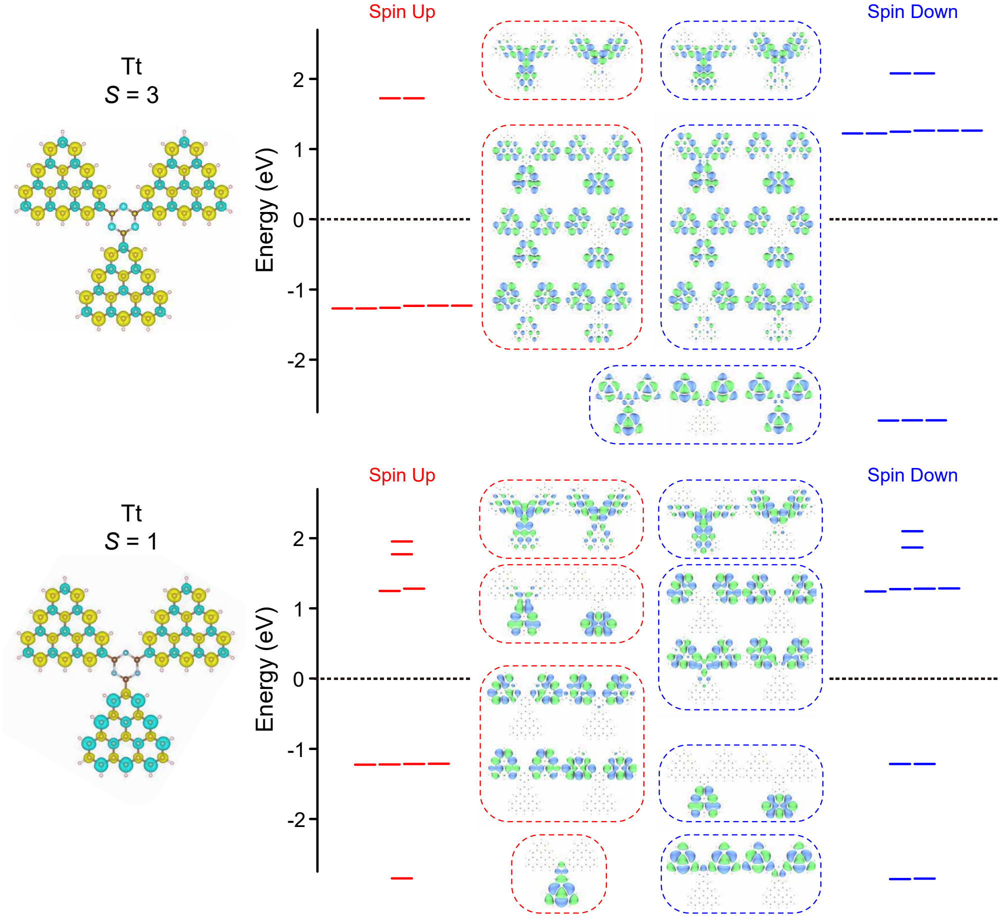

Supplementary Figure 19. DFT calculated spin-resolved energy levels of Tt with S = 1 and S = 3. The calculated spin density distributions are denoted as color green and yellow for spin up and spin down respectively. DFT calculated wave functions of corresponding molecular orbitals are shown beside. The blue and green isosurface colors indicate opposite phases of the wave function.

2.4 Energy levels comparison between spin states of *S* = 3 and *S* = 1 for Tb and Tt

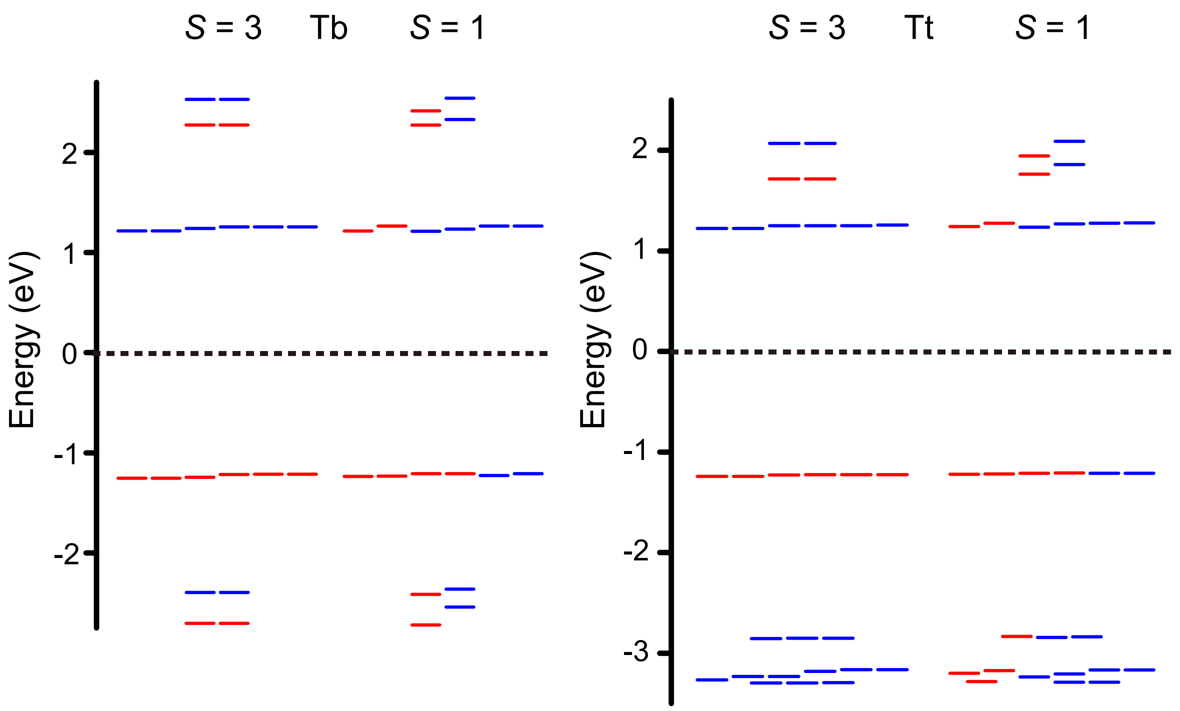

Supplementary Figure 20. DFT calculated spin-resolved energy levels of Tb and Tt in the spin states of *S* = 3 and *S* = 1.

2.5 Theorectical simulated LDOS maps of Tb and Tt in the excited spin state of *S* = 1

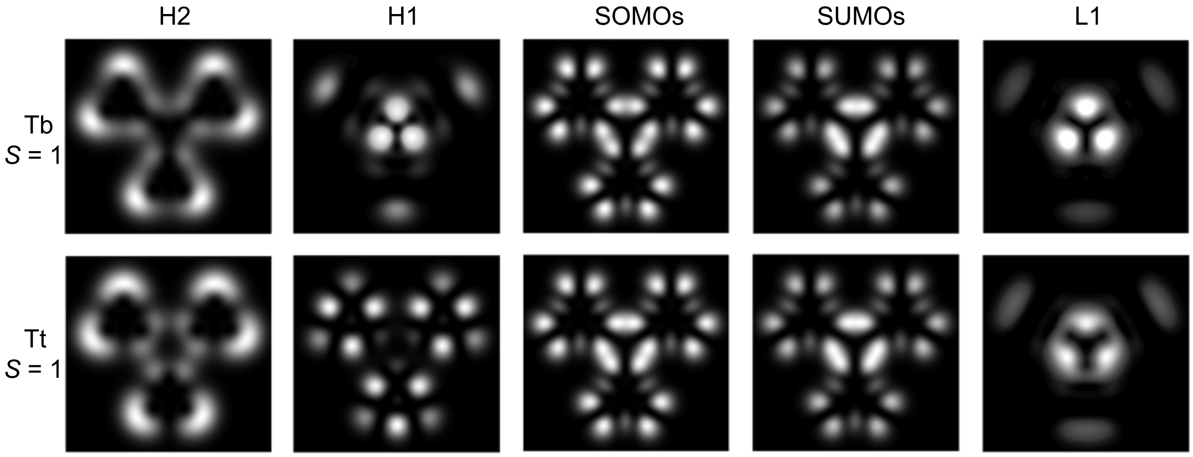

Supplementary Figure 21. Theorectical simulated LDOS maps of Tb and Tt with *S* = 1.

2.6 Constant-height dI/dV maps of Tb and Tt

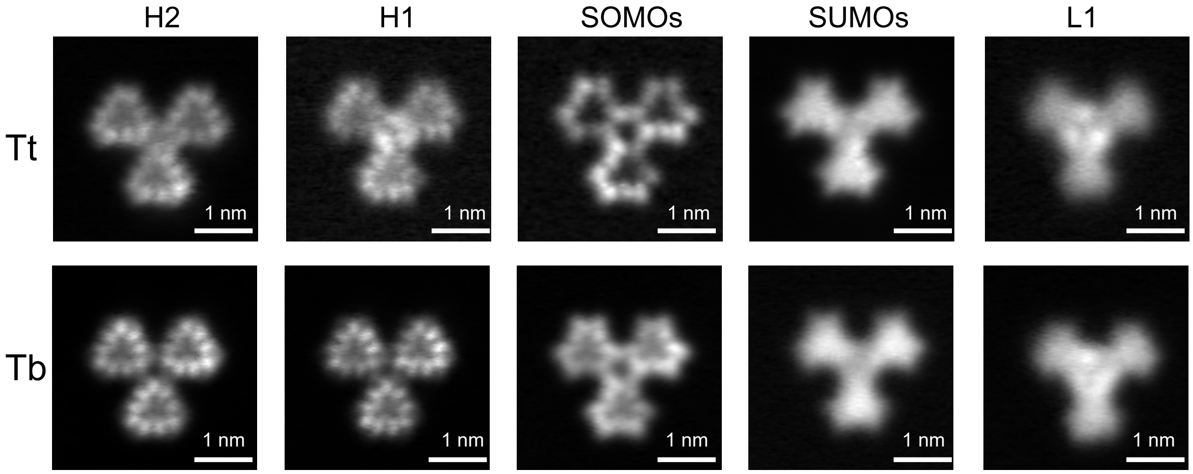

Supplementary Figure 22. Constant-height dI/dV maps of Tb and Tt. Set point: V = 300 mV, I = 30 pA, Δz = -1.5 Å; V_{rms} = 25 mV.

2.7 AFM images of triangulene trimers with additional hydrogen passivation

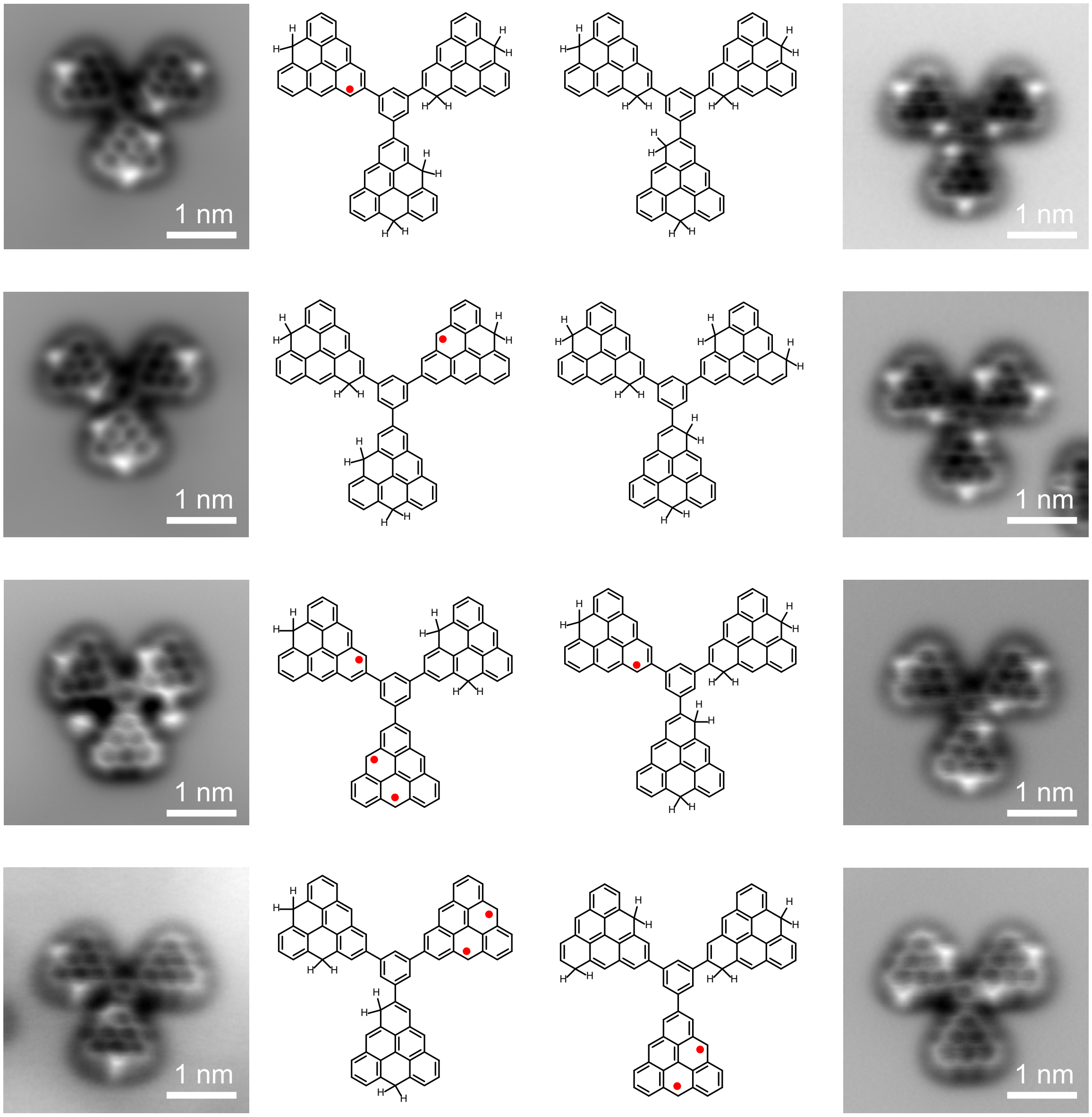

Supplementary Figure 23. AFM images of triangulene trimers with hydrogen passivation. The corresponding molecular models are presented besides.

Supplementary References

1. Mishra, S., Beyer, D.*et al.* Collective all-carbon magnetism in triangulene dimers. *Angewandte Chemie International Edition* **59**, 12041–12047 (2020).

2. Dolomanov, O.V., Bourhis, L.J., Gildea, R.J., Howard, J. A.K. & Puschmann, H. OLEX2: A Complete Structure Solution, Refinement and Analysis Program. *Journal of Applied Crystallography* **42**, 339-341 (2009).

3. Sheldrick, G.M. SHELXT–Integrated space-group and crystal-structure determination. *Acta Crystallographica Section A: Foundations and Advances* **71**, 3-8 (2015).

4. Sheldrick, G.M. Crystal structure refinement with SHELXL. *Acta Crystallographica Section C: Structural Chemistry* **71**, 3-8 (2015).
